# ‘For a wager a hundred yards’: Sir John Floyer’s controlled trial of the effect of cold water upon athleticism (1702)

**DOI:** 10.1177/09677720251368681

**Published:** 2025-08-26

**Authors:** Max Cooper, Sarah Cooper

**Affiliations:** 1 Department of Primary Care and Public Health, 12190 Brighton and Sussex Medical School, Brighton, UK; 2 Department of Neurology Royal Sussex County Hospital, Brighton and Hove, Brighton, UK

**Keywords:** Asthma, coldwater bathing, pulse, fair comparison, Lichfield, Chinese medicine, population measurement

## Abstract

Sir John Floyer (1649–1734) was an English physician and author, notable for developing the pulse watch, advancing understanding of asthma pathophysiology, the first pathological description of emphysema and as an advocate of coldwater bathing. In 1702, he published a book with Edward Baynard MD (c1641–1717) on coldwater bathing. Therein, a postscript to a letter by Baynard describes a controlled trial of water therapy using two boys of ‘near of a Speed and Strength’ to race 100 yards. After immersing the loser in cold water, the race should be re-run. Now the immersed loser emerges as victor. Textual evidence suggests that its author was Floyer rather than Baynard. That the experiment took place is implied by Floyer's certainty in its outcome. It may have served as an ‘exhibition’ to promote his coldwater treatment. Floyer does not appear to repeat his controlled trial in his other books. He does, however, record basic observational comparisons of the effects of age, sex, diet, and month upon pulse rate. He also places data in basic tables and recognises normal variability (‘latitude’) in pulse rate. By linking ranges of pulse rate to clinical outcome, Floyer's work envisions modern clinical decision-making tools.

## Education

Sir John Floyer (1649–1734, see Image 1) was an English physician and author, notable for developing the pulse watch, advancing understanding of the pathophysiology of asthma, the first description of emphysema and as an advocate of coldwater bathing. He was born on the 3rd March 1649 at Hints Hall, near Lichfield, Staffordshire.^1^ Aged 15, Floyer matriculated at The Queen's College, Oxford, going on to receive the following degrees: BA (1668), MA (1670), BM (1674), and DM (1680).^1^ In addition to ‘anatomies’,^2^ an Oxford education ‘on the physic line’ in Floyer's day took the following form:

The course consisted in readings in the medical classics and memorizing the aphorisms of Hippocrates and the works of Galen. These were written and were upheld in disputation. There was no clinical teaching or opportunity for practical experience. The more serious students went abroad for this, especially to Padua^3^

Despite obvious shortcomings of this formation, Floyer was fortunate to be in Oxford at the same time as notable minds of the scientific enlightenment:

[Robert] Boyle lodged for much of the time he was in Oxford … in a house in the High Street, opposite The Queen's College… during the time that Floyer was in residence, where Boyle and Robert Hooke (1635-1703) and their collaborators performed the experiments which laid the foundations of respiratory physiology.^2^

## Early influences

Floyer was influenced by Sanctorius (Santorio Santorio, 1561–1636; see Image 2), an interest probably instigated by renaissance medical books brought back from Padua to Lichfield by Floyer's predecessor, Andrew Hewett (c1603–1684).^2^ These books may account for Floyer's focus upon the impact of lifestyle on physiological factors:

Floyer was a great admirer of … Sanctorius…[who] pioneered the study of metabolism. He monitored his own body functions for some thirty years, documenting chang[es] in body-weight after dining, evacuating and exercising, and correlated these variables against his state of health. For his observations he used a special weighing-chair. Floyer describes weighing an asthmatic patient (probably himself), and also a boy of fourteen, ‘after Sanctorius’ manner’. So he must have copied Sanctorius’ weighing machine and used it in Lichfield.^4^

## Early publications

In 1684 Floyer was knighted ‘for civic rather than medical prominence’ by King Charles the Second.^2^ In 1686 he was elected a member of the Philosophical Society of Oxford.^2^ Floyer went on to publish many books, the first of which came in 1687 and was entitled ‘The Touch-stone of Medicines, Discovering the Virtues of Vegetables, Minerals and Animals, by their Tastes and Smells’.^2^ Floyer reveals that his interest in taste arose from tasting the Jesuits’ bark (Cortex peruvianus) at Oxford.^2^ He goes on to conclude the following about herbal medication:

the true physician knoweth its virtues, the manner of its preparation, the suitableness of it to the humour to be connected, and to the constitution of the patients, all of which the quack is ignorant.^2^

In 1698, Floyer published his first book on the subject of coldwater treatment. This was entitled ‘’An Enquiry into the Right Use of and Abuses of the Hot, Cold and Temperate Baths of England’.^5^ It went through multiple versions and aspects of it are considered in more detail later in this article. Floyer was particularly enthusiastic about coldwater bathing. Between 1697 and 1702, he oversaw the establishment of St Chad's Bath near Lichfield.^2^ Despite Floyer's faith in the therapy, it is of historical note that one contemporary diarist records a death as a consequence:

[the death of a clerical friend] was chiefly owing to an infirmity caused by throwing himself, about midnight in winter time, into the river, upon reading Sir John Floyer of Cold Baths.^6^

A life-long sufferer from asthma,^4^ one subsequent product of his pen was ‘A Treatise of the Asthma’ (1698). In this he reveals insight into the clinical features and pathophysiology of asthma, scientific contributions that are considered in depth by Sakula.^7^ For example, Floyer makes the following astute deduction: ‘I have assign'd the immediate Cause of the Asthma, to the Straitness [i.e. narrowing], Compression, or Constriction of the Bronchia’.^6^ One further contribution to the understanding of respiratory disease was his description of emphysema at post-mortem examination of a ‘broken winded’ horse.^4^

## The physician's pulse watch (1707)

Floyer became fascinated by Chinese medicine^4^ and was one of the first to publish in English on Chinese health beliefs concerning the pulse and acupuncture.^3^ Perhaps his most remarkable publication was a 1707 book entitled ‘The Physician's Pulse Watch’ (see Image 3).^8^ Here Floyer describes how he directed the production of a novel timepiece – possessing a second hand and a special lever to halt the mechanism – for the accurate calculation of pulse rate.^4^ Others had previously attempted to calculate pulse rates by time-consuming and impracticable means. Most notable amongst them before Floyer was Sanctorius’ timepiece known as the ‘pulsilogium’:

Santorio was a pioneer in the use of quantification and developed several types of instruments, among which was a device called pulsilogium that represents the first instrument of precision in the history of medicine. First mentioned in 1602 by a colleague of Santorio in Padua, the instrument possibly constituted a source of inspiration for Galileo and sparked an entire path of experiments in seventeenth-century Europe.^9^

Although the ‘pulsilogium’ broke new ground in physiological measurement, its size and process of calculation appear cumbersome and unsuitable for the busy physician. Floyer notes that others went on to calculate pulse rates, most notably William Harvey (1578–1657). Harvey evidently calculated pulse rate over 30 minutes, a period not convenient for clinical consultations:

This is Dr Harvy's [sic] Computation, the Heart makes a Thousand Pulses in half an hour, and sometimes two Thousand, three Thousand, or four Thousand…^8^

That Harvey's baseline calculation equates to 33 beats per minute reveals the challenges in reaching an accurate reading. It is, however, more accurate than the assertion of Gideon Harvey (senior, c1637-1702) that in the case of ‘an amorous consumption… the heart strikes five hundred sorts of Pulses in an hour’.^10^ All this serves as a reminder of the challenges of accurately calculating the pulse before Floyer. His contribution ­– in contrast with Sanctorius ­– was the introduction of a *practical* device for use in consultations with patients. The concept key to Floyer's timepiece was the recent inception of the second hand, i.e. offering a means to measure seconds:

Thomas Tompion (1639-1713), at Hooke's behest, made a watch for Charles II in 1675, which operated under the control of a spring balance. Though the watch was neither very accurate nor reliable, for the first time in England, the construction of timepieces that showed the passing of seconds as well as of hours and minutes had become technically feasible.^2^

Through this he was able to make important observations, not least the resting pulse rate: ‘the Pulse does not run frequently below sixty’.^8^ He was also the first to record an approximate ratio between pulse and respiratory rate.^2^ The gestation of his timepiece, however, was not straightforward and Floyer outlines the steps involved:

I have for many years tried pulses by the minute in common watches and pendulum clocks, when I was among my patients. After some time I met with the common sea minute-glass, which I used for my cold bathing, and by that I made most of my experiments. But because that was not portable, I caused a pulse watch to be made which run 60 seconds, and I placed it in a box to be more carefully carried, and by this I now feel pulses.^8^

Floyer was thus fortunate to live at the time when measuring seconds and minutes ­– rather than hours ­– became possible. Ultimately, this allowed him to be the first physician to incorporate the pulse reading into his routine examinations of patients.^4^ Floyer's book is also of note for including data (pulse rates) in a basic tabular form under categories of age, along with some further information such as age, sex, body habitus and month of year.^7^

## Later life

Included in his book on the pulse watch is a chapter entitled ‘The English Physician's Cabinet’ (p 329).^8^ This offers a fascinating glimpse inside the medicinal products kept by an eighteenth century physician. In a similar vein, Floyer left manuscripts setting down ‘Advice to a young physician’ on the art, study and knowledge of physic.^2^ This also includes a proposal for founding at Oxford a medical college and teaching hospital to serve the poor.^2^

In ‘Medicina Gerocomica’ (1724), Floyer published one of the first books on the medical aspects of aging.^3^ It is of note here that the English Franciscan friar Roger Bacon had published on ‘The cure of old age and preservation of youth’ in the thirteenth century.^11^ It was at Floyer's recommendation that the young Samuel Johnson (1709–1784) travelled to London to receive the Royal touch of Queen Anne as treatment for the ‘evil’.^3,4^ In 1724, Floyer bequeathed to The Queen's College, Oxford, his collection of books and unpublished manuscripts, including his ‘Advice to a Young Physician’.^2^ Floyer lived to old age such that Johnson noted how he ‘panted on to ninety’.^4^ Floyer was buried at Lichfield Cathedral.^1,2^

Appraisals of Floyer's contributions to medicine are somewhat conflicting. One biographer summarises his contribution by comparison with the great Scottish surgeon John Hunter (1728–1793): ‘Floyer resembled John Hunter in his passion for experiment but lacked his genius in interpretation and orderly thinking’.^3^ A more sympathetic appraisal, however, emphasises how Floyer's enthusiasm laid a solid foundation for others:

He was unquestionably a physician of remarkable energy, curiosity, and intellect, with a fascination for experimental investigation. He was admittedly gullible, and his enthusiasms sometimes obscured his judgment. But we should not underestimate a man who paved the way for the application of measurement to clinical medicine, gave the first description of emphysema, and wrote the first book on medical aspects of old age^4^

## Collaboration with Edward Baynard MD

Floyer published one book with the physician and poet Edward Baynard MD (1641–1717).^12^ Entitled ‘Psykhroloysia: or, The History of Cold Water Bathing’, it was first published under this title in 1702.^13^ The book passed through six editions and this article considers the 1702 edition as it is the first version to include the brief experiment described below.

While Baynard may have been born in Preston, Lancashire,^14^ a search of online parish records revealed only one such ‘Edward Baynard’ born in or close to 1641. This Edward (‘filius Edward Baynard’) was born on the 5th August 1641 at Calne, Wiltshire, England.^14^ In 1670, Baynard was awarded an MA from King's College, Aberdeen, in 1671, matriculated at Leiden, and in about 1672 received an MD from Aberdeen.^1^ He went on to practise medicine in Preston, Bath and London.

## The description of a controlled trial of coldwater

This brief experiment appears in the first edition of Floyer's book on coldwater bathing (see Image 4).^7^ Content from the book had been published in 1697 under the title ‘An Enquiry into the Right Use and Abuses of the Hot, Cold and Temperate Baths in England’.^5^ The experiment, however, does not appear in that edition (which was authored by Floyer alone).^5^ The experiment forms part of the second half of the 1702 book, i.e. a section which ­– at first sight ­– appears to have been written by Baynard. The relevant portion states:

That Cold Water concenters the Spirits and strengthens the Nerves and musculous Fibres, by bracing them, as it were, like a Drum, when the Parchment-head is relax'd, is very evident by this Experiment of two Boys running for a Wager a hundred Yards, more or less, let the Boys be near of a Speed and Strength, take the Boy that loseth and dip him in Cold Water, and then let them run a second time, and the losing Boy shall beat the other, etc.^12^

The writer then offers an account of another observation that appears to have inspired the experiment. It is of note as it lacks the intentional controlled comparison of two athletes:

And talking on this Subject, I remember that a Gentleman told me, That when he was a School-Boy, they used to lay a Twig on two forked Sticks parallel to the Ground, and to jump over, and he said that he always observed, that when he had been in the Water, he could then Spring much higher than at any other time.^12^

The experiment sits within a postscript to a letter written by Baynard. It is not certain whether Baynard or Floyer contributed this part. We deduce, however, this postscript to belong to Floyer's pen for the following reasons. First, is a reference to Floyer in the very first line of the postscript: ‘I had (Sir John)…’.^12^ Second, is its content relating to the health of men in older age. Third, is a mention of a timepiece, namely that bathers ‘wound up their watch, and set their Pendulum in status quo…’.^12^ Fourth, is a reference to Chinese medicine. Finally, there is an impression that the postscript complements [Baynard's] letter by adding further information and referring to ‘our cold regimen’.^12^

That the race took place is strongly implied by its simplicity and Floyer's certainty in its outcome. The experiment is brief but elegant. It offers three key strengths. First, he seeks to make a fair comparison by choosing participants ‘near of a Speed and Strength’.^12^ This description of the selection of similar participants is reminiscent of Lind's 1747 scurvy study where he famously sought to have participants ‘as similar I could have them’.^15^ Second, he establishes a baseline in the form of a comparative order. Third, he reverses this order through a single intervention.

## A link with China?

It is just possible that Floyer's interest in Chinese medicine might lie behind his trial of coldwater treatment on running speed. That is because the Chinese literature includes a broadly similar comparison of two runners. In the eleventh century Ben Cao Tu Jing described the following controlled trial:

It was said that in order to evaluate the effect of genuine Shangdang ginseng, two persons were asked to run together. One was given the ginseng while the other ran without. After running for approximately three to five li [equivalent to 1500 to 2500 meters], the one without the ginseng developed severe shortness of breath, while the one who took the ginseng breathed evenly and smoothly.^16^

## Does Floyer repeat his experimental method during his enquiry into pulse rates?

Floyer's running experiment raises the question of whether he employed the same elegant method elsewhere. This question is particularly relevant to his pulse watch for (with historical hindsight) it opened up a whole world of opportunities for undertaking comparative trials. Floyer provides some evidence of comparisons in pulse rate before and after physical interventions, almost certainly with himself as the subject:

After half an Hours moderate Walking, in a Minute I have counted 112 Pulses; so that the Pulses was [sic] accelerated 20 or 30 Strokes in a Minute, and it fell again not long after the Exercise ceas’d; and before Dinner I counted but 70 Beats in a Minute; but after Dinner they were 90, before the Exercise in the Morning the Pulse was 76.^8^

Floyer appears to have been the first to make these basic observations. He also provides evidence of direct comparison of a single variable, in this case sex:

In November the Pulse of a healthful young Man was 70, of a young Woman 69 in a Morning.^8^

He goes on to make a comparison between two individuals but describes multiple variables. These include diversity in age, sex and bodily habitus:

A fat Man by Walking had 90 Pulses in a Minute, a thin Girl had a 110 by the same Walk, tho’ the Morning Pulse was 75.^8^

Floyer's most experimental observations lie in his presentation of pulse rates categorised by month of the year for ‘healthful’ participants of the same age and sex. Likewise, he contrasted pulse rates in pregnant women who gave birth to a boy with those carrying a girl (but presents no data).^6^ Floyer does not just consider altered pulse rate but also the duration for which such changes are sustained. Here he makes descriptive comparisons between interventions:

‘The alteration of the Pulse by Exercise soon cease, those from Wine and Diet last longer…'^8^

‘Wine changes the Pulses sooner than the solid Meats… this Alteration ceases sooner than that from solid Meats…’^8^

Floyer provides a summary of his findings about the pulse. Here he helpfully categorises interventions or conditions by whether they increase or decrease the pulse rate. Floyer uses these to offer evidence of a relationship between pulse rate and conditions that were considered to be ‘hot’ or ‘cold’ in nature:

By our Pulse we know, that hot Air, hot Cloths, and hot Baths, and hot Meats, and fermented Drinks, plentiful eating, variety of Meats, of bitter Acrid, salt faetid Tastes, or Aromatics, as well as Watching, stopping of Excretions and hot Passions, heat and over-rarifie both our Bloods and Animal Spirits, and consequently accelerate our Pulses; and on the contrary by deficient Pulses we know that cold Baths, cold Diet, Rest, Evacuations, much Sleep, cold Cloths, and Air, and the Passions of Sadness and Fear, sink the Pulse…^8^

His book includes what appears to be the first population-based measurement of a physiological variable: ‘I took the Pulse of 13 Alms-Men in the latter end of May, some [pulse rates] were 91, but the most about 80’.^8^ Likewise, he notes pulse rates of women in hospital. In both cases, Floyer omits the denominator, i.e. size of the overall population.

One issue is that Floyer links pulse rate to variables which today are considered to be fanciful, for example the phase of the moon. Floyer proposes further observational experiment to explore one of these, namely his suspicion that normal pulse rate varied with distance from the equator:

if a Journal were kept of some healthful Pulses every Morning by the Minute-Glass, thro’ a Voyage from England to the Aequator [sic], this Difficulty [question] would be easily decided.^8^

## Floyer's wider clinical observations relating to pulse rate

Nearly all of Floyer's enquiry into pulse rate explores the effect of lifestyle, bodily types or climate. This is probably a reflection of his interest in the research of Sanctorius and contemporary beliefs about disease causation. Floyer emphasizes the importance of clinicians’ learning to recognise a *normal* pulse: ‘We must get a right idea of a moderate Pulse as to Strength and Celerity [rapidity], by often feeling of the Pulse of healthful People…’.^8^ Likewise, he notes a normal rate of 140 bpm for ‘Childrens, whose Pulse are naturally very frequent’.^8^ Finally, he recognises that there is a normal range (‘latitude’) in pulse rates:

I found these varieties of Pulses in a Boy of Eleven, 69, 70, 72, 76. By this ‘tis evident, That young Boys have a Latitude in their healthy Pulses.^8^

It is surprising that Floyer does not undertake experiments into the effects of different plants upon pulse rate. Instead, he considers other clinical contexts, for example the following comment on the effect of bloodletting:

I observ'd the Pulse of a thin Woman in the Cholick by eating Fruit 100 in a minute; the Pain rais’d the Fever, but the pulse sunk much after Bleeding.^8^

He then deduces indications for bloodletting: ‘We know when Bleeding is proper by the Pulse when ‘tis high, full, strong, quick, and very frequent’.^8^ He also recognises the value of examining the pulse in preventing excessive bleeding: ‘all that time we must feel the Pulse, and observe the Stream for fear of killing the Patient by excessive Bleeding’.^8^

Finally, it is of note that his ‘Table of the pulses according to Diseases’^8^ offers an embryo of today's clinical decision tools by linking pulse to clinical outcome. The examples below only illustrate three of his categories:

The Number of Pulses

140. THIS is the most exceeding Pulse, such as happens in Dying Persons… and we find it in Fits of Fevers… [Floyer then offers clinical explanations for reducing pulse ranges, of which two brief examples are]...

Pulses from 50 to 55. The undose [sic], weak Pulse, after great Evacuations without a Fever, as Haemorrhagies, Diarrheas, sweats, Diabetes’

Pulses from 45 to 40. Here by Analogy I conjecture the dying pulse may be plac'd…^8^

## Discussion

Here we present a summary of the biographies of Sir John Floyer and his co-author Edward Baynard. Floyer's greatest contribution to medicine lies in his introduction of a practical means for measuring the pulse in everyday consultations. This offers insight into the challenges of introducing new technology into clinical medicine and resonates with the adoption of ‘bedside tests’ in modern times.

Today it is impossible to imagine a consultation about a physical health problem without measuring the pulse rate. Floyer's timepiece meant that he was the first to make this routine in his consultations. His interest in the pulse appears to have been inspired by Sanctorius and a fascination with Chinese medicine. Indeed, Floyer notes how Chinese doctors used to assess a patient's pulse for 30 minutes.^8^

The conception of his timepiece was not without problems and the following account will strike a chord with today's doctors whose medical equipment still requires annual calibration (see Figures 1 to 4):

Since [my pulse] Watch does run unequally, rather too fast for my Minute-Glass, I thereby regulate it; and add 5 or 6 to the Numbers told by the Watch.^8^

**Figure 1.**
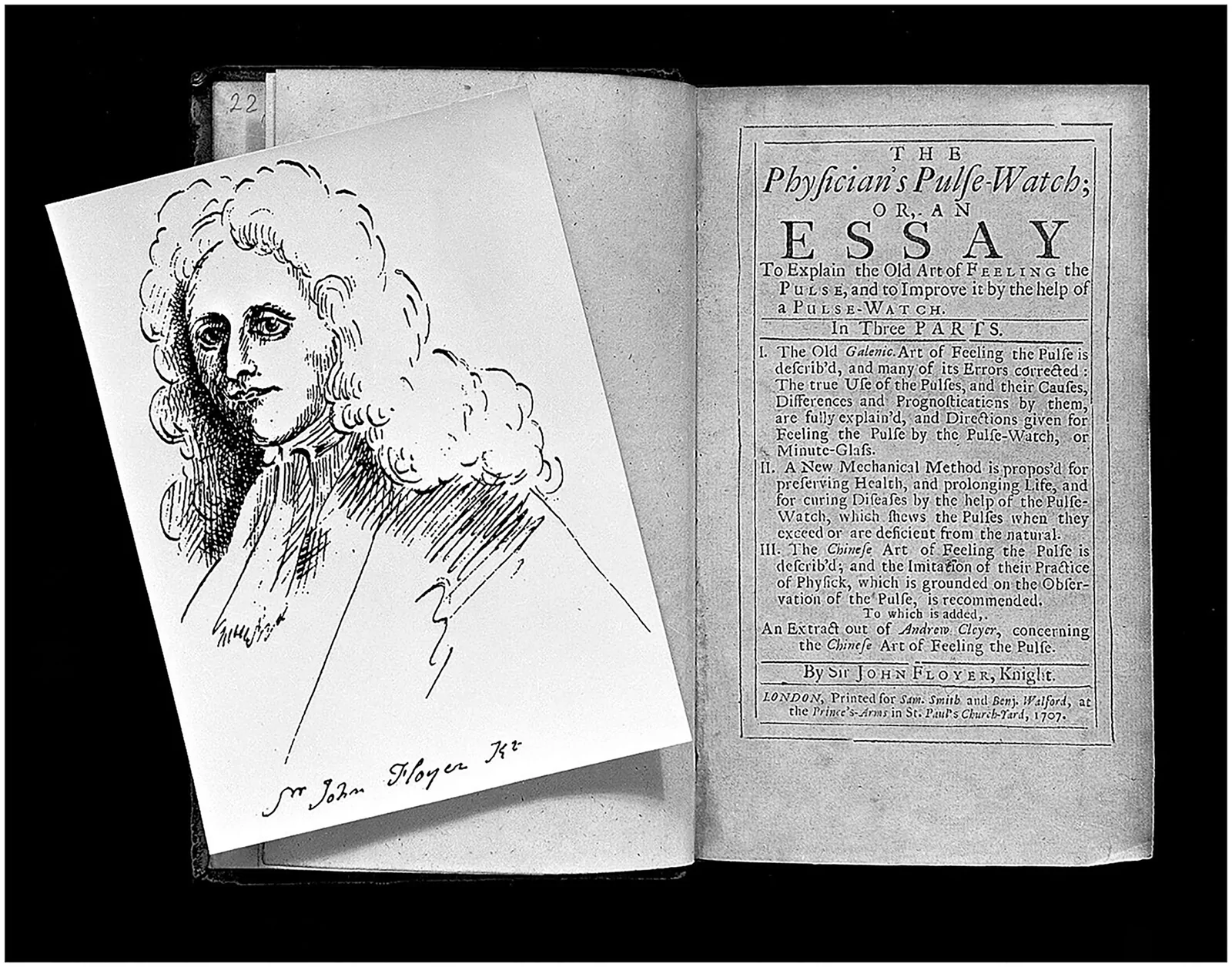
Sir John Floyer (1649–1734). The physician's pulse-watch; or, an essay to explain the old art of feeling the pulse, and to improve it by the help of a pulse-watch…. To which is added, an extract out of Andrew Cleyer, concerning the Chinese art of feeling the pulse (An appendic. I. An essay to make a new sphygmologia … II. An inquiry into the nature … of the respirations … III. A letter concerning the rupture in the lungs). Image courtesy of the Wellcome Collection.

**Figure 2.**
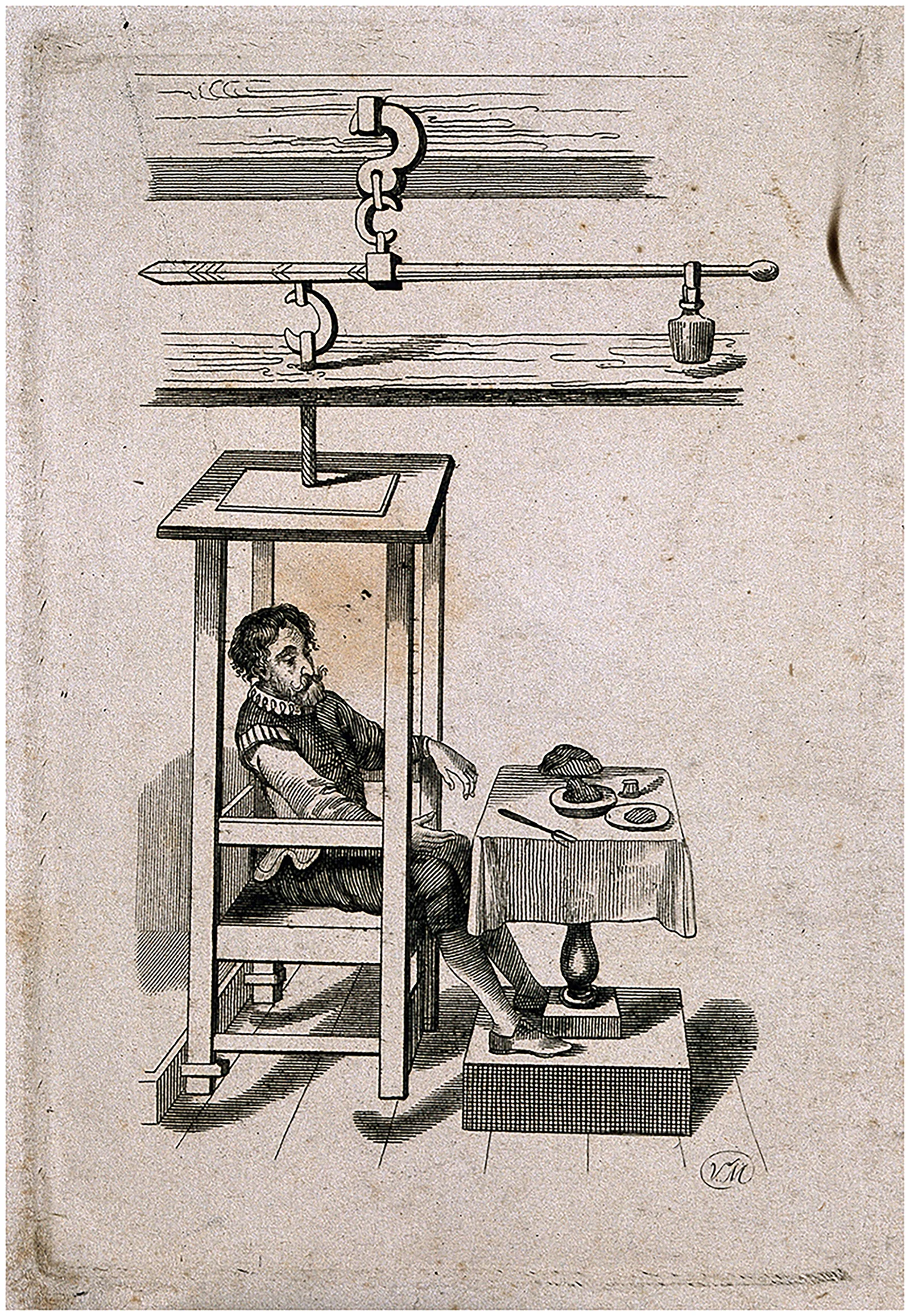
Sanctorius Sanctorius (1561–1636). There is evidence that Floyer replicated this device in Lichfield. It is of historical note that similar technology was common during the Victorian age in the form of ‘jockey scales’ . These were used in country houses, for example at Petworth House in Sussex, England, to match visitors with a suitable stead when riding. Image courtesy of the Wellcome Collection.

**Figure 3.**
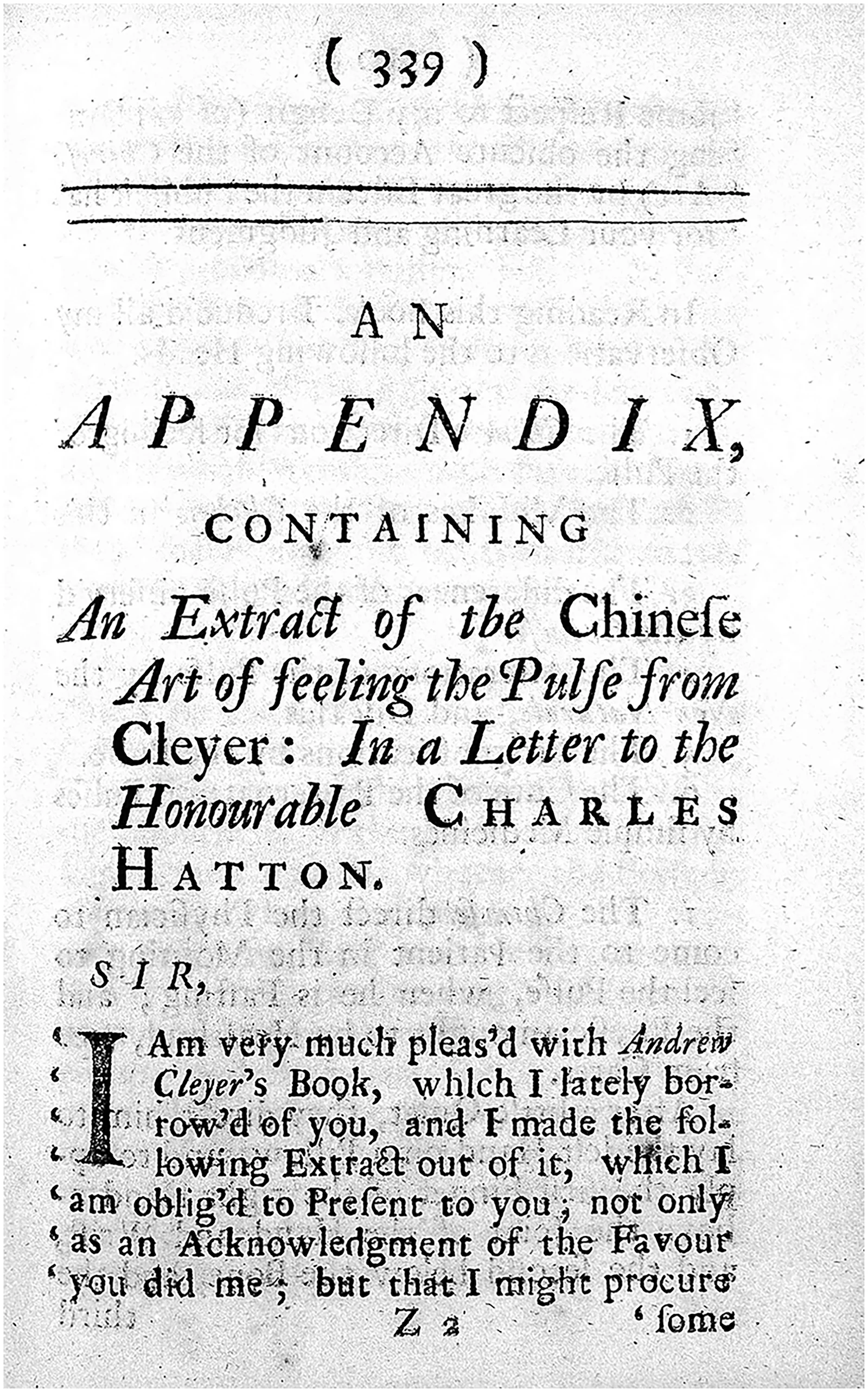
Floyer had a keen in interest in Chinese medicine. This page is from his book The physician's pulsewatch. Floyer notes how Chinese doctors would feel a patient's pulse for thirty minutes. Image courtesy of the Wellcome Collection.

**Figure 4.**
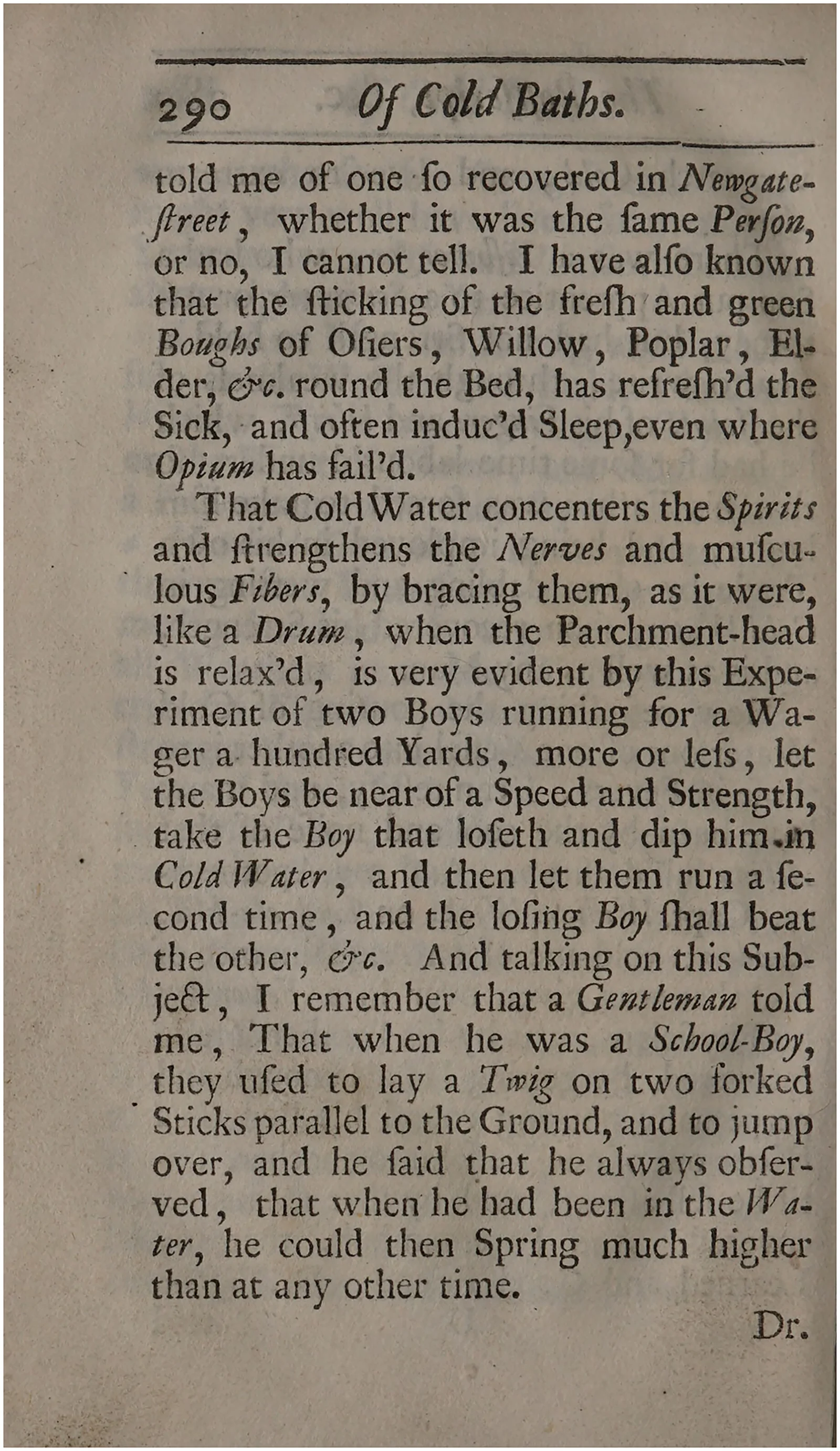
Description of controlled trial of cold water (1702). From The ancient psykhroloysia revived: or, an essay to prove cold bathing both safe and useful. In four letters…. Also a letter of Dr Baynard's, containing an account of many eminent cures done by the cold baths in England. Image courtesy of the Wellcome Collection.

One overlooked portion of his 1702 book on coldwater bathing lies in a very brief but elegant description of a controlled trial. Floyer seeks to link this controlled evidence with (outdated) theory of the ‘bracing’ of fibres. Given his other medical interests, it seems ironic that Floyer did not attribute it to increased pulse rate or opening of the airways. We submit that this controlled trial was the work of Sir John Floyer (i.e. rather than Baynard). That claim is based on his name's inclusion at the beginning of the postscript alongside references to a timepiece, to China and to ageing. One future solution to this authorship puzzle could lie in the use of digital ‘stylometry’. Through this, the written contribution of an author to a piece can be measured. This has famously been used elsewhere, most notably in identifying Shakespeare's collaborative authorship of the plays Henry VIII (1613) and Two Noble Kinsmen (1634).^17^

It seems clear from his confidence in the outcome that Floyer must have implemented his coldwater experiment at least once. No date for it is offered, although it must have been undertaken in or before 1702 and possibly at St Chad's Bath, Lichfield. Indeed, the trial may have been used repeatedly to persuade clients of the performance-enhancing benefits of Floyer's coldwater treatment. As such, we posit that the race constituted more of an ‘exhibition’ (or even ‘sales pitch’) than a methodological turning point in Floyer's career. This can be deduced from his subsequent enquiries which are all basic observations of nature rather than a ‘decisive experiment’.^18^

That is all despite the fact that Floyer's pulse watch created a world of opportunities for comparative studies along the lines of his coldwater experiment, in particular to compare the effects of herbal medication upon pulse rate. His experimental mind is subsequently most evident in his comparison of pulse rate and the month of year in individuals of the same age and sex. Many of his observations appear to have been undertaken by examining himself. This suggests that engaging patients in clinical research was challenging even in his day. Floyer is ahead of his day by placing data in basic tables^2^ and undertaking what appears to amount to a survey of a population, e.g. of Lichfield alms-men and of women in hospital. Unfortunately, he overlooks what proportion of the population participated but (given the simplicity of the examination) it seems likely that all those who were present partook.

## Conclusion

To modern eyes Floyer's race appears to be a physiological experiment but for him it offered clear evidence of the benefit of a medical treatment. Floyer's experiment describes the phenomenon of a reversal of participant order due to a single intervention. So simple an example of a controlled trial can help medical students understand the concept of a fair comparison. Furthermore, Floyer's biography illustrates for students the practical challenges of introducing new technology in medicine. This has implications for tests and equipment increasingly available today, for example ‘wearable technology’ (watches or phones) that record physiological measurements such as pulse rate or a basic heart tracing. It remains unclear how these are best integrated into primary care to improve patient outcomes as well as to ensure that healthcare resources reach the the most needy. Although Floyer struggled to apply his pulsewatch along the same experimental lines of his coldwater ‘wager’, his approach of basic observation, documentation in tables and recognition of normal offers a sensible starting place.

Floyer lived during the scientific enlightenment and at the dawn of physiological measurement. His comparative study of coldwater is elegant but does not appear to have influenced others who published on controlled trials in the eighteenth century, most notably Hauksbee the Younger^18^ and James Lind.^15^ This is most evident in the fact that Lind does not mention Floyer's trial in his 1753 ‘Treatise of the Scurvy’. ^15^Floyer's legacy has understandably been overshadowed by inspirational physicians and surgeons, especially John Hunter (1728–1793), James Lind (1716–1794), and Edward Jenner (1749–1823). Floyer never pursued the precise impact of his medical treatments on patients. Reason for this appear to lie in a preference for personalised care for private patients, his fondness for lifestyle interventions (especially coldwater treatment) alongside an impression that medication only served an auxillary role in healing:

It is a true saying, That Nature performs the Cure in Diseases, and the Physician only assists her by his Medicines.^8^
